# Identification of plant vacuole proteins by using graph neural network and contact maps

**DOI:** 10.1186/s12859-023-05475-x

**Published:** 2023-09-22

**Authors:** Jianan Sui, Jiazi Chen, Yuehui Chen, Naoki Iwamori, Jin Sun

**Affiliations:** 1 School of Information Science and Engineering, University of Jinan, Jinan, China; 2 Laboratory of Zoology, Graduate School of Bioresource and Bioenvironmental Sciences, Kyushu University, Fukuoka-Shi, Fukuoka, Japan; 3 School of Artificial Intelligence Institute and Information Science and Engineering, University of Jinan, Jinan, China; 4 School of Computer Science and Engineering, University of Electronic Science and Technology of China, Chengdu, 611731 China

**Keywords:** Plant vacuole proteins, Peroxisomal proteins, SeqVec, AlphaFold2, Graph convolutional neural network

## Abstract

Plant vacuoles are essential organelles in the growth and development of plants, and accurate identification of their proteins is crucial for understanding their biological properties. In this study, we developed a novel model called GraphIdn for the identification of plant vacuole proteins. The model uses SeqVec, a deep representation learning model, to initialize the amino acid sequence. We utilized the AlphaFold2 algorithm to obtain the structural information of corresponding plant vacuole proteins, and then fed the calculated contact maps into a graph convolutional neural network. GraphIdn achieved accuracy values of 88.51% and 89.93% in independent testing and fivefold cross-validation, respectively, outperforming previous state-of-the-art predictors. As far as we know, this is the first model to use predicted protein topology structure graphs to identify plant vacuole proteins. Furthermore, we assessed the effectiveness and generalization capability of our GraphIdn model by applying it to identify and locate peroxisomal proteins, which yielded promising outcomes. The source code and datasets can be accessed at https://github.com/SJNNNN/GraphIdn.

## Introduction

Plant vacuoles are unique organelles composed of a monolayer membrane and their internal cell fluid, and are mostly found in plant cells [1, 2]. Plant vacuoles have cell functions such as degradation, autolysis and regulation. They play several important roles in the cell, including storage, waste disposal, and maintenance of turgor pressure. Vacuoles can store a variety of substances, including water, ions, nutrients, and pigments [3]. In recent years, a growing body of evidence has demonstrated the crucial role of the three-dimensional structure of vacuolar proteins in their cellular transport and localization. For instance, studies have shown that the vacuolar sorting receptor 4(VSR4) and vacuolar sorting receptor 6(VSR6) receptors located on the vacuolar membrane can recognize the C-terminal HDEL domain of vacuolar proteins, thereby facilitating their transportation into vacuoles [4, 5]. And other studies have demonstrated the importance of the three-dimensional structure of the Arabidopsis thaliana vacuolar H +—pyrophosphatase (AVP1) in proper targeting to the tonoplast membrane. Mutations that disrupt the structure of AVP1 have been found to cause mislocalization and reduced activity [6, 7].

To obtain the 3D structures of plant vacuole proteins, the recently developed AlphaFold2 [8] can be employed. AlphaFold2 demonstrates exceptional accuracy in predicting protein structures. According to the Protein Data Bank (PDB) official website (https://www.rcsb.org/), a substantial number of protein structures have been solved. As of September 5, 2023, the Protein Data Bank (PDB) boasts a substantial collection, encompassing around 48,272 resolved plant vacuole protein structures. Additionally, an impressive total of 28,118 structures emerged from computational endeavors through Computed Structure Models (CSM) experiments. Among these predictive models, 6,278 boast confidence scores (pLDDT) exceeding 90, while 15,952 fall within the pLDDT range of 70–90. Additionally, 5,534 models exhibit pLDDT scores ranging from 50 to 70, with only 354 models displaying pLDDT scores below 50. In general, a pLDDT score exceeding 70 serves as a robust indicator of the reliability of a predicted protein structure. Elevated pLDDT scores signify a greater concordance between the predicted protein structure and the actual structure, typically associated with a higher quality prediction. Remarkably, approximately 80% of the determined structures of plant vacuole proteins in the PDB boast pLDDT scores surpassing 70, thereby underscoring the dependability of these predictions. This degree of reliability holds substantial significance for our investigations in plant vacuole protein identification.

Simultaneously, an increasing body of experimental evidence has substantiated the fact that AlphaFold2-predicted protein 3D structures significantly contribute to the process of identification and characterization of various biological entities. Duan et al. [9] discovered that the protein structure predictions generated by AlphaFold2 offer valuable insights into the identification and classification of the A1 aspartate protease family. Specifically, these predictions are particularly informative for the characterization of nucleoprotein-like and atypical members within the family. Cheng et al. [10] utilized the 3D protein structure predictions generated by AlphaFold2 to aid in the identification and functional analysis of members belonging to the tobacco INV gene family. Their study confirmed the utility of the predicted protein structure in unraveling the mechanistic insights into INV function, thereby providing valuable information for a comprehensive understanding of the functional aspects associated with the INV gene family.

Furthermore, elucidating the mechanisms that maintain the biogenesis of vacuoles requires a comprehensive understanding of the biochemical and physiological roles of plant vacuole proteins [2, 11]. However, traditional biological experiments are time-consuming and expensive. Therefore, it is essential to develop efficient computational methods for identifying plant vacuole proteins.

In recent years, various models have been proposed for identifying organelle proteins. In the field of Golgi protein identification, researchers have employed different feature extraction and prediction methods to achieve high accuracy. For example, Ahmad et al. [12] utilized a combination of split amino acid composition (SAAC), 3-gap dipeptide composition (3-gap DPC), and bigram position-specific scoring matrix (Bigram PSSM) as feature extraction methods, achieving an accuracy of 94.8% in identifying Golgi proteins. Zhou et al. [13] proposed a new Golgi protein type prediction method that combined pseudo amino acid composition (PseAAC), dipeptide composition (DC), pseudo position-specific scoring matrix (PsePSSM), and an ensemble of binary classifiers by evidence-based group work (EBGW) to extract feature vectors. They selected extreme gradient boosting (XGBoost) as the classifier, and the best accuracy of the model reached 92.1%. Lv et al. [14] developed a Golgi protein localization classifier called isGP-DRLF, which evaluated ten widely-used machine learning algorithms, finding that the best independent test accuracy was 98.4%. Moreover, other models have been developed for identifying Golgi proteins [15–20]. In the field of mitochondrial protein identification, researchers have used various features and classifiers to predict the sub-mitochondrial localization of proteins. For example, Du and Li [21] carried out the first study on the identification and localization of sub-mitochondrial proteins. Lin et al. [22] used the highly representative tetrapeptide selected by binomial distribution to predict the sub-mitochondrial position of mitochondrial proteins and generated the dataset M317. The prediction accuracy of support vector machine (SVM) as a classifier reached 94%. Additionally, there are several other models that have been developed to identify mitochondrial proteins, such as those described in references [23–28]. In addition, Anteghini et al. [29] developed the In-Pero model in 2021 to identify peroxisomal proteins. This model utilized the deep learning embedding methods UniRep [30] and SeqVec [31] to extract the properties of peroxisomal proteins. The authors reported a high accuracy of 92% for identifying peroxisomal proteins using the In-Pero model, as determined by cross-validation. However, there are few tools available for identifying plant vacuole proteins. Yadav et al. [32] proposed a prediction model called VacPred for identifying plant vacuole proteins. The VacPred model uses the SVM algorithm and two classical feature extraction methods: dipeptide combination (DPC) and k-spaced position-specific scoring matrix (K-PSSM), a feature descriptor based on the position-specific scoring matrix (PSSM). The VacPred model achieved independent test accuracy of 86.49% and fivefold cross-validation accuracy of 81.75%. Jiao et al. [33] developed an efficient plant vacuole protein prediction model called iPVP-DRLF by using the deep learning embedding model UniRep [30] to extract features, and applying a two-step feature selection strategy involving the combination of light gradient boosting machine (LGBM) and sequential forward search (SFS) to identify the optimal feature subset from each high-dimensional feature. iPVP-DRLF achieved fivefold cross-validation and independent test accuracy values of 88.25% and 87.16%, respectively, which were better than the previous state-of-the-art prediction values.

**Fig. 1:**
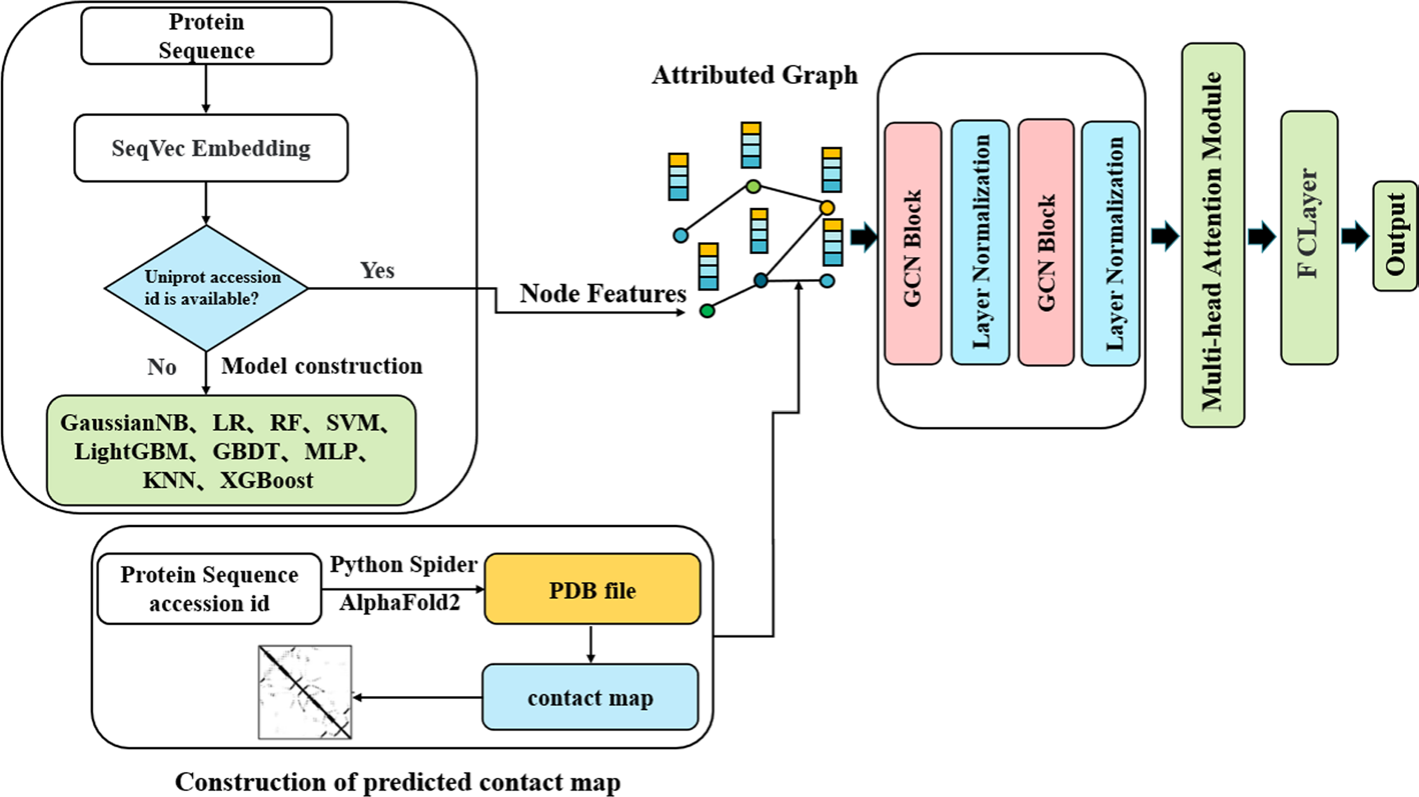
The overall framework of the GraphIdn model

The current tools for identifying plant vacuolar proteins are limited, and previous studies on plant vacuoles have mostly relied on protein sequences, ignoring the structural information of proteins. To address this, we developed a plant vacuole protein recognition model called GraphIdn. We incorporated the structural information of plant vacuole proteins using the AlphaFold2 algorithm. To obtain PDB files containing the structural information, we input protein accession numbers into the AlphaFold2 website (https://alphafold.ebi.ac.uk/). However, downloading PDB files one by one was not scalable, so we developed a Python crawler program to automate the process. By inputting multiple protein sequence accession numbers into a text file, our crawler program could download the corresponding PDB files from the AlphaFold2 website in bulk. Once we obtained the PDB files, we calculated the corresponding contact maps and fed them into a graph neural network. The structural features obtained from the graph neural network were then inputted into a multi-head attention module and finally a fully connected layer to identify plant vacuole proteins. The node features of the graph neural network were initialized using the deep representation learning model SeqVec, which was trained on the protein sequences. The overall GraphIdn model flow is illustrated in Fig. 1.

## Materials and methods

### Datasets

#### Dataset of vacuole proteins

**Fig. 2:**
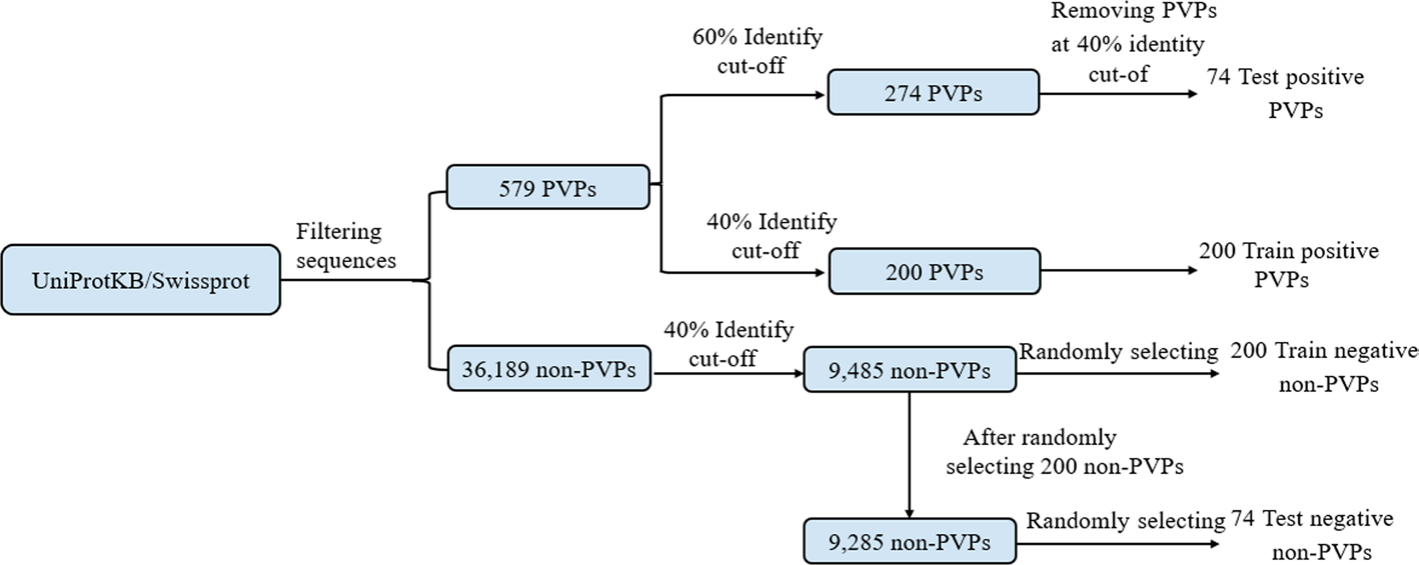
Flow chart of vacuole proteins datasets construction

The selection of an appropriate and accurate dataset is a critical step in the model training process and has a significant impact on the model's accuracy. In this study, we used the dataset collected by Yadav et al. [32]. They searched the UniprotKB/SwissProt database [34], removed sequences with nonstandard amino acids and identified a total of 579 plant vacuole proteins (PVPs) and 36,189 non-plant vacuole proteins (non-PVPs). Among the 579 plant vacuole proteins, the CD-HIT [35] program was applied, resulting in 200 and 274 protein sequences at the 40% and 60% identity cutoffs, respectively. Subsequently, 200 protein sequences were chosen from the 40% identity cutoff group as positive samples for the training dataset. To construct an independent dataset, they established distinction by implementing cut-offs at 60% (274) and 40% (200) levels for proteins, employing these as the independent positive dataset. Similarly, Yadav et al. employed CD-HIT to identify 9,485 protein sequences from a pool of 36,189 non-plant vacuole proteins using a 40% identification cutoff. To establish dataset balance, they conducted multiple rounds of random selection [36] to choose 200 protein sequences from the initial pool of 9,485 sequences. Subsequently, the top-performing 200 protein sequences were utilized as negative samples for the training dataset. In a similar manner, 74 protein sequences were randomly selected multiple times from the remaining pool of 9,285 sequences, and the best performing 74 protein sequences were then chosen as negative samples for the independent test set. The construction of the dataset is depicted in Fig. 2. Table 1 presents the number of proteins in the dataset.

**Table 1:** Protein distribution in the dataset of vacuole proteins

Categories of proteins	Number of proteins
PVPs	274
Non-PVPs	274

#### Dataset of peroxisome proteins

In this study, we employed the dataset for peroxisomal proteins created by Anteghini et al. [29] in 2021. They conducted a search in the UniprotKB/SwissProt database to collect 327 sequences of peroxisomal membrane proteins. Applying the CD-HIT program, they selected 162 protein sequences at the 40% identity cutoff. Next, they further refined their selection to include only proteins with at least one relevant publication-specific subcellular localization, resulting in 132 highly curated sequences of peroxisomal membrane proteins.

Similarly, a search in the UniprotKB/SwissProt database provided 60 peroxisomal matrix protein sequences. After applying the CD-HIT program, they obtained 22 protein sequences at the 40% identity cutoff. They further narrowed down their selection to include only proteins with at least one relevant publication-specific subcellular localization. This screening process yielded 19 highly curated peroxisomal matrix protein sequences.

**Fig. 3:**
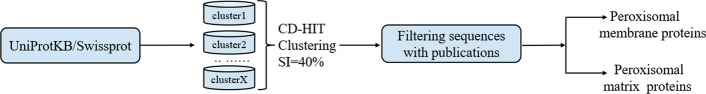
Flow chart of peroxisome proteins dataset construction

Due to the limited number of matrix proteins obtained, they conducted another search in the Uniprot protein database and obtained 721 peroxisomal matrix protein sequences. Applying the CD-HIT program, they selected 202 protein sequences at the 40% identity cutoff. Further screening based on proteins with specific subcellular localization in at least one relevant publication led to 22 highly curated peroxisomal matrix protein sequences. Combining these two subsets resulted in a total of 41 peroxisomal matrix protein sequences, from which 13 common entries were removed, ultimately leaving 28 unique peroxisomal matrix protein sequences. The basic construction process of the peroxisome protein dataset is shown in Fig. 3. Table 2 presents the number of proteins in the dataset.

**Table 2:** Protein distribution in the dataset of peroxisomal proteins

Categories of proteins	Number of proteins
Membrane	132
Matrix	28

### Protein contact maps

The protein contact map is a concise representation of a protein's structure, presented as a symmetrical two-dimensional matrix. The dimensions of the matrix correspond to the number of residues in the protein sequence. The matrix elements are binary, specifically '1' or '0', indicating whether there is a contact or absence of contact between the residues. The prevailing global standard for defining protein contact maps adheres to the authoritative criteria set forth by the International Critical Assessment of Protein Structure Prediction (CASP) [37]. According to this definition, when assessing whether two residues within a protein structure are in contact, the Euclidean distance between their ${C}_{\beta }$ atom (for glycine, it is the ${C}_{\alpha }$ atom) is pivotal. If this distance is less than 8 Å, the residues are deemed to be in contact. Conversely, if the Euclidean distance exceeds 8 Å, it signifies that the two residues are not in contact. In order to obtain contact maps of proteins, we used AlphaFold2. AlphaFold2 is a deep learning-based algorithm developed by DeepMind for protein folding prediction. It uses a neural network to predict the 3D structure of a protein from its amino acid sequence. The network was trained on a large dataset of known protein structures using a two-stage approach. In the first stage, the network predicts the distance between pairs of amino acids. In the second stage, the network uses this distance information to predict the 3D structure of the protein. The network is trained using a combination of supervised and unsupervised learning techniques. The AlphaFold2 algorithm also uses a novel attention mechanism to help the network focus on the most relevant parts of the protein when predicting its structure. This attention mechanism is similar to the one used in natural language processing to allow neural networks to focus on different parts of a sentence.

### Feature extraction

#### Amino acid embedding

In prior research, the methods used to extract features from protein sequences were primarily based on traditional coding techniques, such as manual features derived from component features, location features, and physical and chemical properties. However, these methods ignore a significant amount of information that is hidden between residues in the sequence. Recently, deep representation learning models have been applied for protein sequence representation [38–42]. These models were trained on a large database of protein sequences and represented the protein sequence as a continuous vector using a deep embedding model. In this study, we employed the SeqVec model, which leverages the deep bidirectional ELMo model commonly used in natural language processing, to acquire the vector representation of the protein sequence. ELMo models the protein sequence as a probability distribution and integrates evolutionary information into the embedding, effectively capturing the biophysical properties of protein sequences from a large database (UniRef50).

Each protein sequence is first converted to an integer sequence according to the following function:

1 $$f(m_{j} ) = i$$

2 $$i = 1,2.......,20,\;\;if\;m_{j} \in {2}0{\text{ canonical amino acid}}$$

where ${m}_{j}$ is the *j* th amino acid of the sequence. The integer sequence $f({m}_{j})$,j = 1, 2, 3, 4, ……L (length of protein sequence) is embedded into 1024-long feature vectors via the model named SeqVec.

#### Structural feature extraction

The protein space graph is defined as *G* = (*V, A*), where *V* represents the set of nodes. For the amino acid node feature *X* of a protein sequence of length* L*, we initialize the amino acid sequence using the model named SeqVec. *X* ∈ ${R}^{L\times D}$, *D* represents the feature dimension, which is 1024 dimensions.* A*∈${R}^{L\times L}$ represents the adjacency matrix, which is calculated from the contact map and can describe the position between two residues in the space. The GCN module in our model consists of two GCN layers, each of which can be described by the following formula:

3 $$H^{(l + 1)} = \sigma (\mathop D\limits^{{ \sim }{ - \frac{1}{2}}} \mathop A\limits^{ \sim } \mathop D\limits^{{ \sim }{ - \frac{1}{2}}} H^{(l)} W^{(l)} )$$

where $\mathop A\limits^{ \sim }$ = $A + I$,$I$ is the unit matrix. $\mathop D\limits^{ \sim }$ is a diagonal degree matrix of $\mathop A\limits^{ \sim }$.$H$ is the feature of each layer, for the input layer $H$ is *X*. *W* is the weight matrix of a specific layer of trainable parameters.$\sigma$ is a nonlinear activation function, we use the ReLU function. In order to accelerate the convergence of the GCN layer, there is a normalization layer behind each GCN layer that maps its output to the range of [0,1]. The output of the final GCN layer is the feature matrix *M*, *M* ∈ ${R}^{L\times o}$,*o* represents the output dimension of the GCN layer. The dimension of *M* is related to the length of the amino acid sequence. In order to eliminate the sequence alignment variance and the size variance [43] to obtain a fixed representation, we use the multi-head attention mechanism:

4 $$T = softMax(W_{2} \tanh (W_{1} M^{T} ))$$

$T$ $\in$ ${R}^{k\times L}$, *k* is the number of attention groups. The *k* groups of attention coefficients assess the contributions of each amino acid to the identification of plant vacuole proteins from different perspectives.${W}_{1}$, ${W}_{2}$ are two learned attention matrices with hyperparameters* k* and *f*,${W}_{1}\in {R}^{f\times o}$,${W}_{2}\in {R}^{k\times f}$. Finally, we multiply the matrix *M* and *T* as the output of our multi-head attention module.

### Feature selection

Since the plant vacuole protein sequence features extracted by SeqVec model may have redundant information, it is easy to affect the performance of the model. Herein, we employ the elastic regression network (Elastic Net) as a feature selection method to identify the optimal protein feature set. Elastic Net is a regularization technique that combines both ${L}_{1}$ and ${L}_{2}$ regularization. The ${L}_{1}$ regularization imposes sparsity by setting some of the coefficients to zero, while the ${L}_{2}$ regularization controls the magnitude of the non-zero coefficients. The Elastic Net algorithm balances these two regularization terms to achieve both sparsity and accuracy.

Mathematically, the Elastic Net algorithm can be expressed as follows:

5 $$\min ||y - X\beta ||^{2} + \lambda_{1} ||\beta ||_{1} + \lambda_{2} ||\beta ||_{2}^{2}$$

where *y* is the response vector, *X* is the feature matrix,* β* is the coefficient vector, ${\lambda }_{1}$ and ${\lambda }_{2}$ are the regularization parameters that control the ${L}_{1}$ and ${L}_{2}$ penalties, respectively.

By varying the values of ${\lambda }_{1}$ and ${\lambda }_{2}$, Elastic Net can select the optimal subset of features that can predict the response variable with high accuracy. To apply Elastic Net for protein feature selection, we first constructed a feature matrix containing all the candidate protein features, and then performed Elastic Net regression to identify the optimal subset of features. The selected features were used as inputs for our machine learning models. Overall, the use of Elastic Net as a feature selection method enabled us to identify the most informative features of plant vacuole proteins while avoiding overfitting and improving the predictive performance of our models.

### Traditional machine learning classifier

We constructed and evaluated multiple traditional machine learning classifiers to identify plant vacuole proteins using nine classification algorithms that have previously been used for similar applications. The employed algorithms include gaussian naive bayes (GaussianNB), logistic regression (LR), random forest (RF), support vector machine (SVM), light gradient boosting (LightGBM), gradient boosted decision trees (GBDT), multilayer perceptron (MLP), k-nearest neighbors (KNN), and extreme gradient boosting (XGBoost). Gaussian naive bayes (GaussianNB) is a simple and fast algorithm for classification tasks. It is a probabilistic algorithm based on Bayes' theorem and assumes that the features of a data point are independent and normally distributed. Logistic regression (LR) is a commonly used statistical method for binary classification tasks. It is a linear model that uses a logistic function to predict the probability of a data point belonging to one of two classes. Random forest (RF) is an ensemble machine learning algorithm for classification and regression tasks. Support vector machine (SVM) is a supervised learning algorithm for classification and regression tasks. It works by finding the hyperplane in high-dimensional space that best separates the data into classes. Light gradient boosting machine (LightGBM) is a gradient boosting framework that uses tree-based learning algorithms. Gradient boosting decision tree (GBDT) is an ensemble learning method that uses a combination of decision trees to make predictions. Multilayer perceptron (MLP) is a type of artificial neural network used for supervised learning. K-nearest neighbor (KNN) is a simple and widely used machine learning algorithm for classification and regression. Extreme gradient boosting (XGBoost) is an optimized and scalable implementation of the gradient boosting algorithm for decision tree-based learning. These algorithms were implemented through the scikit-learn [44], and we fine-tuned their hyperparameters through grid search to achieve the best possible performance. In this study, we fed feature vectors of plant vacuole proteins into different algorithms and compared their performance to select the most effective one.

### Fully connected layer

Fully connected layers are a type of neural network layer commonly used in deep learning models. These layers are typically positioned towards the end of the network and are responsible for mapping the output from the preceding layers to a set of output classes. In the GraphIdn model, the protein spatial structure features obtained by the graph convolutional neural network are inputted into the multi-head attention module, followed by a fully connected layer that identifies organelle proteins. The matrix is transformed into an m-dimensional vector, where m is the number of organelle protein types, using the fully connected layer. As the identification of plant vacuole proteins and peroxisomal proteins in this study is a binary classification task, the value of m is 2. The SoftMax function is then applied to map the values to the interval [0, 1], and the plant vacuole proteins and peroxisomal proteins are identified based on the maximum index of the output two-dimensional matrix.

### Evaluation metrics and methods

Accuracy (Acc), sensitivity (Sn), specificity (Sp), Matthews correlation coefficient (MCC) and F1-score were utilized to evaluate the performance of the prediction system [45–50]. The calculation method is as follows:

6 $$S{\text{p}} = \frac{TN}{{TN + FP}}$$

7 $$Sn = \frac{TP}{{TP + FN}}$$

8 $$Acc = \frac{TP + TN}{{TP + FN + TN{ + }FP}}$$

9 $$F1 = \frac{2 \times TP}{{2 \times TP + FN + FP}}$$

10 $$MCC{ = }\frac{TP \times TN - FP \times FN}{{\sqrt {(TP + FP) \times (TP + FN) \times (TN + FN) \times (TN + FP)} }}$$

In this study, we are examining the identification of plant vacuole proteins, which presents as a binary classification problem with only two potential outcomes (0 and 1). The accuracy of the prediction is determined by four categories: true positive (TP), false positive (FP), true negative (TN), and false negative (FN). The ratio of correct prediction in positive and negative samples is represented by Sn and Sp, respectively. The F1 score measures the robustness of the model, with a higher score indicating a stronger robustness. The overall accuracy of the predictor is reflected by Acc. However, when the data set is unbalanced, Acc may not provide an accurate evaluation of the classification results and it is better to use Matthews Correlation Coefficient (MCC) instead. Additionally, the performance of the model is evaluated using receiver operating characteristic area under the curve (ROC-AUC) and precision-recall area under the curve (PR-AUC). ROC-AUC represents the area under the ROC curve and the higher the value, the better the model. The relationship between precision and recall is depicted by the PR curve, where precision is represented by P and recall is represented by R. In general, recall is set on the x-axis and precision on the y-axis. Similarly, PR-AUC is calculated by measuring the area under the PR curve, with a higher value indicating a better performance of the model.

## Result and discussions

### Performance of fivefold cross-validation and independent experiments on traditional machine learning models

To identify plant vacuole proteins, we first utilized the SeqVec model to convert protein sequences into continuous vectors. We then evaluated the performance of traditional machine learning models on the plant vacuole protein datasets.

It is observed from Table 3 that the highest Acc, F1-score, Sp, Sn, ROC-AUC, PR-AUC and MCC values of the relevant models on the independent test set are 66.89%, 0.6839, 75.67%, 71.62%, 0.7144, 0.7190 and 0.3394, respectively. The LightGBM model is found to be the best overall performer, outperforming other models in terms of Acc, MCC and ROC-AUC indicators. The GaussianNB model performs the worst, with an accuracy of only 59.46%.

**Table 3:** The performance of traditional machine learning models on the independent test set

Model	Acc (%)	F1-score	Sp (%)	Sn (%)	MCC	ROC-AUC	PR-AUC
GaussianNB	59.46	0.6386	47.30	71.62	0.1950	0.6291	0.7190
LR	63.51	0.6582	56.76	70.27	0.2728	0.6888	0.6570
SVM	66.89	0.6839	62.16	71.62	0.3394	0.7014	0.6796
RF	66.22	0.6667	64.86	67.57	0.3244	0.7062	0.7001
LightGBM	66.89	0.6573	70.27	63.51	0.3386	0.7144	0.7074
GBDT	64.18	0.6345	66.22	62.16	0.2840	0.6843	0.6763
MLP	62.16	0.6164	63.51	60.81	0.2433	0.6770	0.6639
KNN	60.14	0.6144	56.75	63.51	0.2032	0.6462	0.6600
XGBoost	63.51	0.5846	75.67	51.35	0.2786	0.6707	0.6681

Table 4 illustrates the results of the evaluation of traditional machine learning models in the fivefold cross-validation. The LightGBM model is found to have the best performance, with the highest Acc, F1-score, Sp, Sn, ROC-AUC, PR-AUC and MCC values of 65.23%, 0.6868, 70.43%, 67.85%, 0.6504, 0.8100 and 0.3838, respectively. The LightGBM model outperforms other models in terms of Acc, F1-score, Sp, Sn, and MCC. Meanwhile, the MLP model have the worst performance, except for the Sp index, as its other indicators are lower than those of other models.

**Table 4:** The performance of traditional machine learning model on the fivefold cross -validation

Model	Acc (%)	F1-score	Sp (%)	Sn (%)	MCC	ROC-AUC	PR-AUC
GaussianNB	60.00	0.6163	54.46	65.02	0.1971	0.6096	0.7010
LR	58.25	0.5791	58.45	57.80	0.1625	0.6141	0.6099
RF	60.75	0.5980	63.07	58.44	0.1503	0.6127	0.5868
SVM	54.50	0.5678	58.96	55.99	0.2166	0.5925	0.5427
LightGBM	65.23	0.6868	70.43	67.85	0.3838	0.5809	0.5429
GBDT	57.25	0.6569	67.38	65.05	0.3253	0.5751	0.5754
MLP	52.50	0.4934	57.43	47.07	0.0447	0.5650	0.5462
KNN	56.50	0.5189	59.02	49.68	0.0869	0.5722	0.8100
XGBoost	56.50	0.5015	64.28	45.89	0.1043	0.6504	0.8100

Subsequently, we employed the Elastic Net model as a feature selection approach to identify the most relevant and informative features, thereby eliminating any redundant features that may have been present. The resulting optimal feature set was further reduced to 175 dimensions and subsequently utilized as input for nine traditional machine learning models for comprehensive analysis and evaluation.

**Fig. 4:**
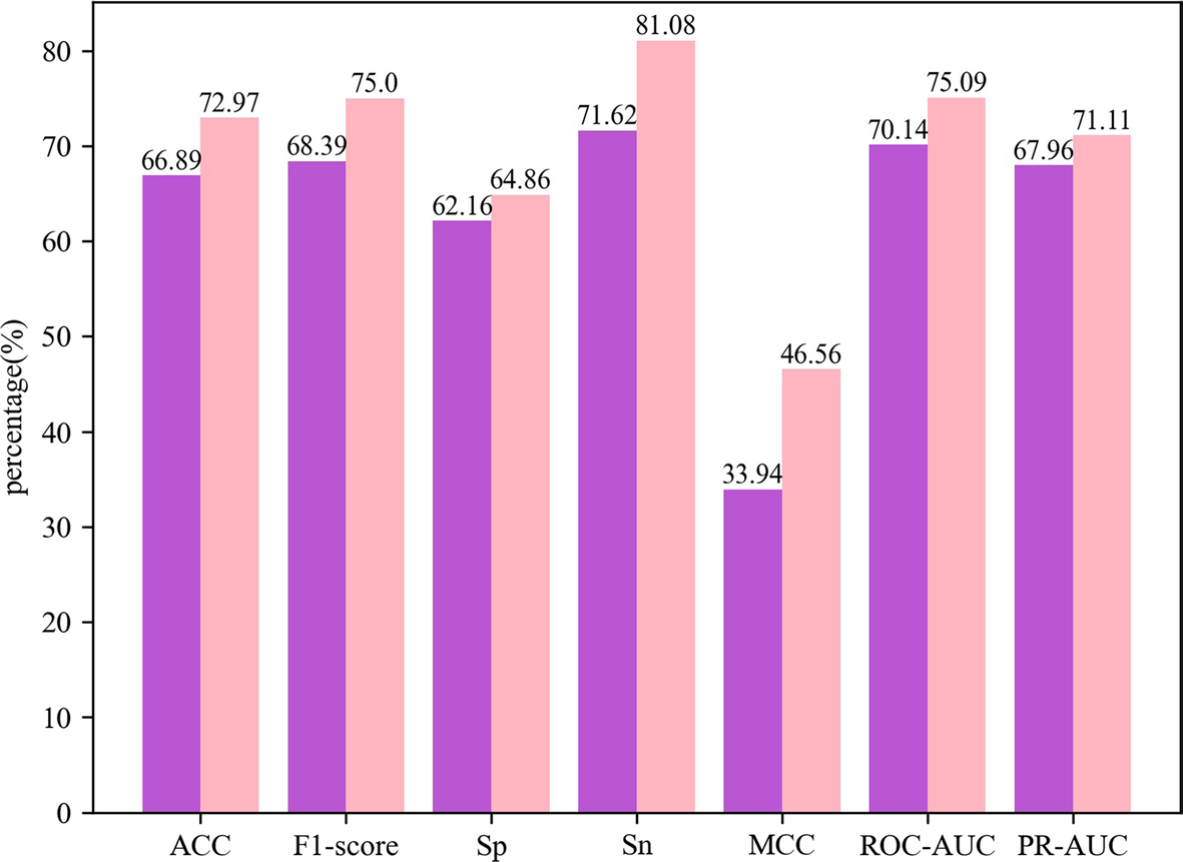
The performance of the LR model on the independent test set before and after feature selection

**Fig. 5:**
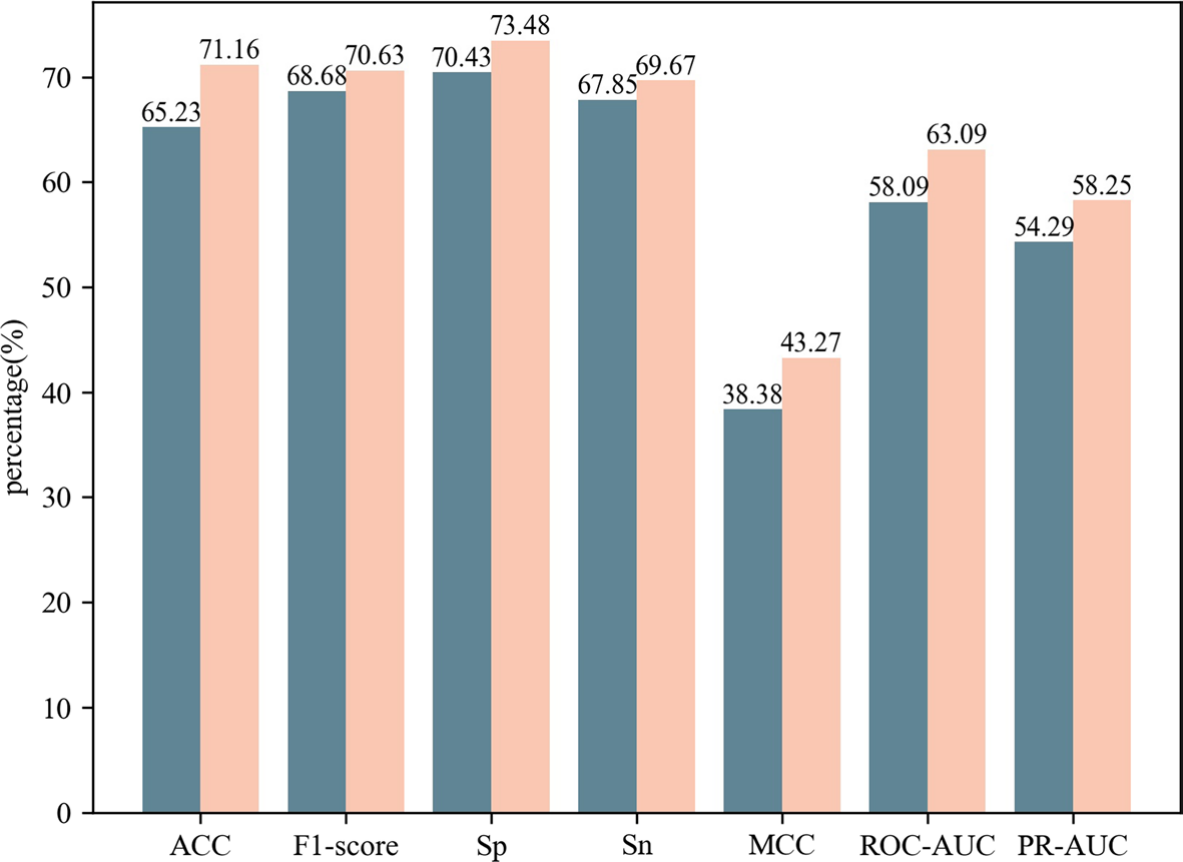
The performance of the LightGBM model on the fivefold cross-validation before and after feature selection

The performance of the machine learning models improved significantly after the feature selection process using the Elastic Net model. The largest improvement was seen in the LR model, which had a 9% increase in accuracy, reaching 72.97%. Figure 4 compares the best-performing LR model before and after feature selection. After feature selection, the performance of each model improved on the fivefold cross-validation compared to before feature selection. The Acc value of the LightGBM model increased to 71.16%, which was roughly 6% higher than its previous value. Figure 5 shows the comparison between the best-performing LightGBM model before and after feature selection.

### Performance of fivefold cross-validation and independent experiments on the GraphIdn model

In this study, we utilized the AlphaFold2 algorithm to obtain the structural information of plant vacuole proteins in our dataset and subsequently calculated contact maps. These contact maps were then used as inputs for a graph convolutional neural network. The structural features obtained from the graph neural network were then fed into a multi-head attention module and finally into a fully connected layer, which was utilized to identify plant vacuole proteins.

**Fig. 6:**
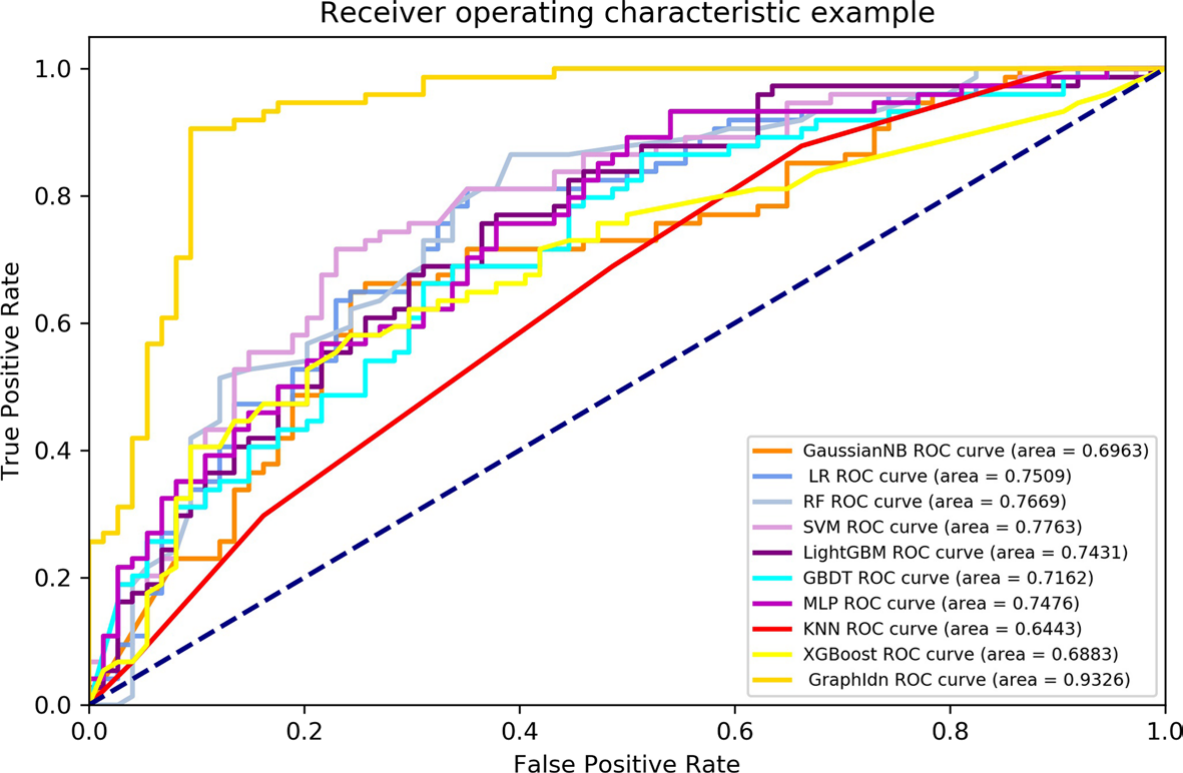
ROC curve of models on the independent test set

**Fig. 7:**
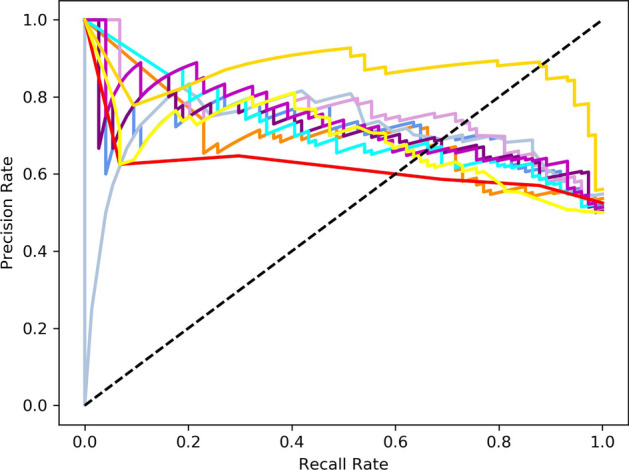
PR curve of models on the independent test set

As shown in Table 5, we compare our GraphIdn model with previous models after feature selection on the independent test set. The results of the independent test set show that the GraphIdn model has an Acc of 88.51%, F1-score of 0.8917, Sn of 82.43%, Sp of 94.59%, MCC of 0.7760, ROC-AUC of 0.9326, and PR-AUC of 0.9140. These results indicate that the GraphIdn model outperforms the best overall performing LR model by around 15.6% in terms of accuracy rate and has a higher Matthews correlation coefficient by 0.31 when compared to the LR model. Additionally, the F1-score, Sp, Sn, ROC-AUC, and PR-AUC values of the GraphIdn model are around 0.142, 17.6%, 13.5%, 0.182, and 0.203 higher, respectively, compared to those of the LR model. Figures 6 and 7 also show the ROC and PR curves of each model on the independent test set.

**Table 5:** The performance of models on the independent test set

Model	Acc (%)	F1-score	Sp (%)	Sn (%)	MCC	ROC-AUC	PR-AUC
GaussianNB	63.51	0.6667	54.05	72.97	0.2752	0.6963	0.6971
**LR**	**72.97**	**0.7500**	**64.86**	**81.08**	**0.4656**	**0.7509**	**0.7111**
RF	71.62	0.7308	66.22	77.03	0.4350	0.7669	0.7560
SVM	69.59	0.6939	70.27	68.92	0.3919	0.7763	0.6976
LightGBM	68.24	0.6846	67.57	68.92	0.3649	0.7431	0.7100
GBDT	64.86	0.6667	59.46	70.27	0.2990	0.7162	0.7128
MLP	64.87	0.6338	68.92	60.81	0.2983	0.7476	0.7339
KNN	60.14	0.6335	51.35	68.92	0.2059	0.6443	0.6186
XGBoost	65.54	0.5641	86.49	44.59	0.3423	0.6883	0.6824
**GraphIdn**	**88.51**	**0.8917**	**82.43**	**94.59**	**0.7760**	**0.9326**	**0.9140**

**Fig. 8:**
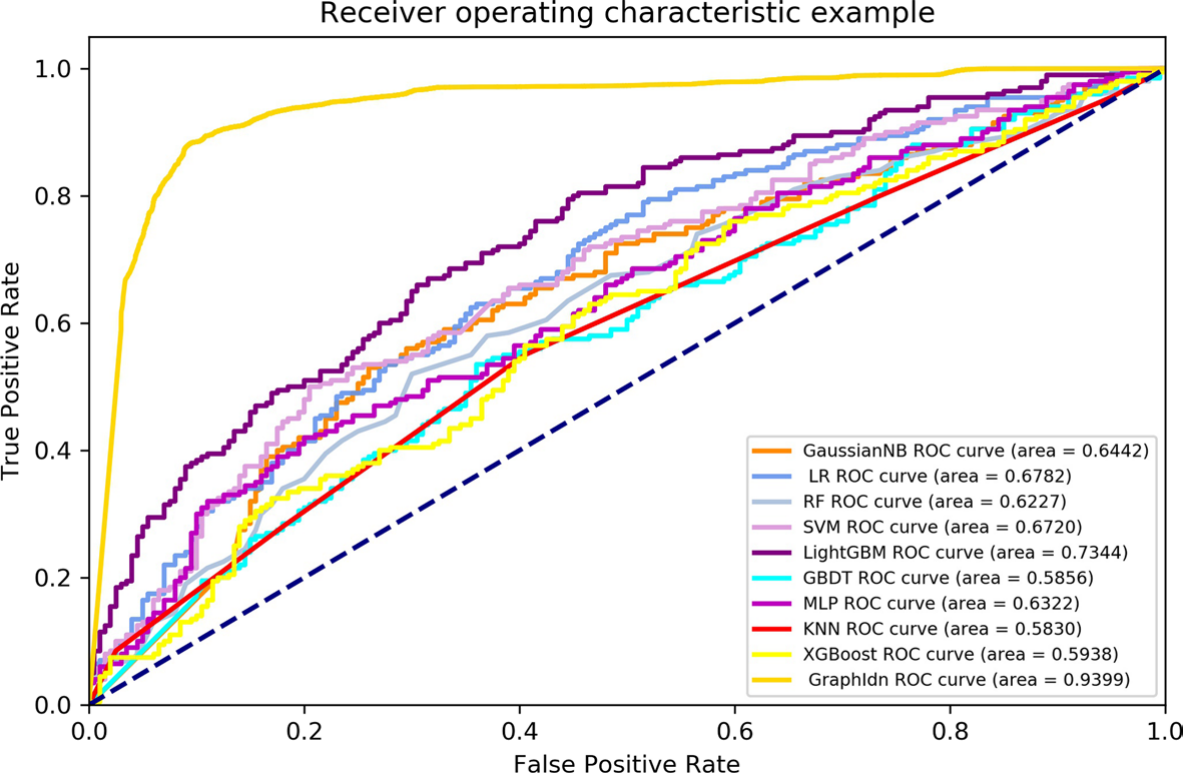
ROC curve of models on the fivefold cross-validation

**Fig. 9:**
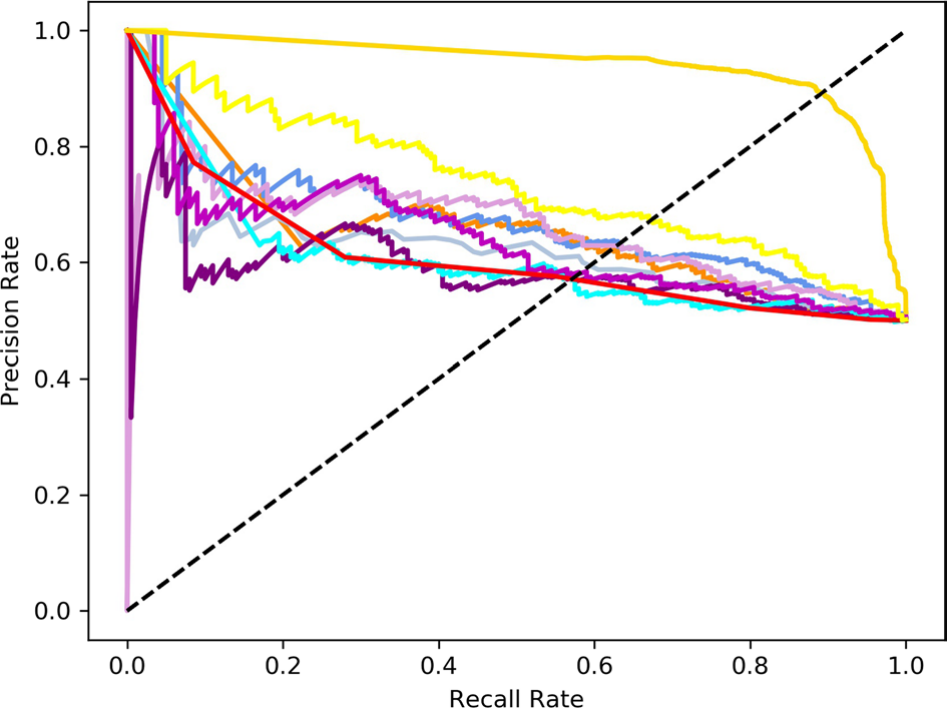
PR curve of models on the fivefold cross-validation

At the same time, our model was also tested on the fivefold cross-validation. As shown in Table 6, we compare the GraphIdn model with previous models after feature selection on the fivefold cross-validation. The Acc, F1-score, Sp, Sn, MCC, ROC-AUC, and PR-AUC values of the GraphIdn model on fivefold cross-validation are 89.93%, 0.8917, 89.70%, 90.47%, 0.8020, 0.9399, and 0.9191, respectively. These values are 18.1% higher than the accuracy of the best overall performance model, LightGBM. Additionally, the F1-score, Sp, Sn, MCC, ROC-AUC, and PR-AUC values of the GraphIdn model are around 0.185, 16.2%, 20.8%, 0.370, 0.206, and 0.337 higher, respectively, compared to those of the LightGBM model. As shown in Figs. 8 and 9, we also draw the ROC curve and PR curve of each model on the fivefold cross-validation. The results of the fivefold cross-validation and independent test set experiments demonstrate that our model outperforms traditional machine learning models. Furthermore, the efficacy of the AlphaFold2 structural model is validated through a comparative assessment of experimental outcomes between employing protein sequences represented by the SeqVec model as direct inputs into conventional machine learning models and utilizing GraphIdn, which incorporates structural information.

**Table 6:** The performance of models on the fivefold cross-validation

Model	Acc (%)	F1-score	Sp (%)	Sn (%)	MCC	ROC-AUC	PR-AUC
GaussianNB	62.00	0.6289	58.30	65.11	0.2355	0.6442	0.6553
LR	62.75	0.6307	61.61	63.97	0.2554	0.6782	0.6724
RF	60.25	0.5835	64.62	56.20	0.2104	0.6227	0.6165
SVM	62.75	0.6002	64.57	58.46	0.2316	0.6270	0.6527
**LightGBM**	**71.16**	**0.7063**	**73.48**	**69.67**	**0.4327**	**0.7344**	**0.5825**
GBDT	58.00	0.6689	70.75	65.62	0.3649	0.5856	0.6127
MLP	58.00	0.5730	58.26	57.32	0.1570	0.6322	0.6372
KNN	57.25	0.5659	58.89	56.12	0.1506	0.5830	0.7388
XGBoost	61.00	0.5636	63.21	54.19	0.1757	0.5938	0.7388
**GraphIdn**	**89.93**	**0.8917**	**89.70**	**90.47**	**0.8020**	**0.9399**	**0.9191**

Bolded values are the models that perform better

Finally, to investigate the usefulness of AlphaFold2 structure models, we have devised two models: the first utilizes the contact graph, generated from the structural insights provided by the AlphaFold2 model, as the adjacency matrix. The second model, in contrast, does not rely on the AlphaFold2 structural model. Instead, we construct the adjacency matrix ourselves arbitrarily. Within the second scenario of not utilizing the AlphaFold2 structural model, we have subdivided it into three distinct models: one with a randomly constructed adjacency matrix, another with an adjacency matrix consisting solely of ones, and a third with an adjacency matrix comprising exclusively of zeros. As such, we have designed a total of four models to meticulously assess the practical applicability of the AlphaFold2 structural models. Table 7 distinctly indicates that the performance of the GraphIdn models, which exclude the utilization of AlphaFold2 structural models, demonstrates notably inferior results when compared to the GraphIdn models utilizing the AlphaFold2 structural models. Particularly, this distinction becomes more pronounced, especially when the adjacency matrix comprises solely zeros. This outcome strongly underscores the utility of AlphaFold2 structural models.

**Table 7:** The impact of AlphaFold2 structural models on the performance of the GraphIdn

Model	Adjacency matrix	Acc (%)	Sn (%)	Sp (%)	MCC	ROC-AUC
**GraphIdn (With structural features)**	**With topology**	**88.51**	**94.59**	**82.43**	**0.776**	**0.933**
GraphIdn (Without structural features)	Random construction	85.10	91.89	77.03	0.722	0.917
	All 1	83.11	90.54	75.67	0.670	0.914
	All 0	50.00	0.00	100.0	0.00	0.490

Bolded values are the models that perform better

We then proceeded to compare the performance of our proposed GraphIdn model with a recently proposed model, IPVP-DRLF [33], and previously proposed models, VacPred-DPC [32] and VacPred-PSSM [32], as shown in Tables 8 and 9. All these models were trained using the identical dataset as the GraphIdn model, and the same independent test set was employed for evaluation. They have been published in high-quality journals and their experiments and model parameter adjustments are the best results available. Additionally, Yadav et al. [32] developed more than 30 different types of models and finally selected two models with the best performance, including one dipeptide composition-based and one PSSM-based model. In the proposed iPVP-DRLF, Jiao et al. [33] used 12 feature extraction methods for comparative experiments and finally selected the best-performing method.

**Table 8:** Comparison of GraphIdn model with previous models on the independent test set

Model	Acc (%)	Sn (%)	Sp (%)	MCC	ROC-AUC
VacPred-DPC	80.41	82.43	78.38	0.610	0.840
VacPred-PSSM	86.49	90.54	82.43	0.730	0.930
iPVP-DRLF	87.16	89.19	85.14	0.744	0.916
**GraphIdn**	**88.51**	**94.59**	**82.43**	**0.776**	**0.933**

Bolded values are the models that perform better

**Table 9:** Comparison of GraphIdn model with previous models on the fivefold cross- validation

Model	Acc (%)	Sn (%)	Sp (%)	MCC	ROC-AUC
VacPred-DPC	75.50	70.00	81.00	0.510	0.800
VacPred-PSSM	81.75	76.50	87.00	0.640	0.860
iPVP-DRLF	88.25	89.00	87.50	0.765	0.933
**GraphIdn**	**89.93**	**90.47**	**89.70**	**0.802**	**0.940**

Bolded values are the models that perform better

Our model outperformed the other models in terms of accuracy, sensitivity, Matthews correlation coefficient and ROC-AUC values on the independent test set, improving by 1.35%, 5.40%, 0.032, and 0.017 respectively. On the fivefold cross-validation, our model achieved higher accuracy, specificity, sensitivity, Matthews correlation coefficient, and ROC-AUC values, enhancing by 1.68%, 2.20%, 1.47%, 0.037, and 0.007, respectively. These results demonstrate the superior performance of our model.

Finally, we assessed the impact of pLDDT on the performance of our GraphIdn model by partitioning the independent test set into two subsets based on pLDDT scores, specifically pLDDT > 70 and pLDDT < 70. Remarkably, the ratio of samples possessing pLDDT > 70 to those with pLDDT < 70 stands at an approximate proportion of 4:1 within the independent test set. Subsequently, we compared the accuracy discrepancy between these two subsets. The outcomes of this experimentation are presented in Table 10. The findings indicate that the model's accuracy in the pLDDT > 70 subset of the independent test set is approximately 4.8% higher compared to the pLDDT < 70 subset. Moreover, all other performance metrics also exhibit improvements in the pLDDT > 70 subset compared to the pLDDT < 70 subset. This observation underscores that a higher pLDDT score corresponds to more reliable predictions from our model, thus leading to enhanced experimental outcomes.

**Table 10:** The effect of pLDDT on the experimental results of our GraphIdn model

PLDDT	Acc (%)	Sn (%)	Sp (%)	MCC	ROC-AUC
> 70	85.47	93.22	77.59	0.718	0.930
< 70	80.65	86.66	75.00	0.619	0.913

In order to verify the generalization performance of our model, we also experimented on the dataset for peroxisomal proteins. The AlphaFold2 algorithm was utilized to obtain the structural information of peroxisomal proteins, and then contact maps were calculated and inputted into the graph convolutional neural network to identify peroxisomal proteins. After tenfold cross-validation, the model performance in Acc, F1-score, Sp, Sn, MCC, ROC-AUC, PR-AUC values reached 94.90%, 0.970, 82.27%, 98.60%, 0.8230, 0.9093, 0.9748, respectively. In addition, we also compared with the model named In-Pero proposed in 2021[29]. As shown in Table 11, the Acc, F1-score and MCC values are increased by 3%, 0.111, 0.102, respectively. This experiment further proves the superiority and good generalization performance of the proposed GraphIdn model.

**Table 11:** Performance comparison between GraphIdn model and In-Pero model

Model	Acc (%)	F1-score	MCC
In-Pero	91.9	0.859	0.721
**GraphIdn**	**94.9**	**0.970**	**0.823**

Bolded values are the models that perform better

## Discussion

From the experimental results presented in this paper, it is evident that we have achieved promising outcomes in using protein structure information for the identification of plant vacuole and peroxisomal proteins. This lays the groundwork for future applications of this method in identifying proteins in other organelles. However, our research has certain limitations. Firstly, for the problem of identifying plant vacuole proteins, the performance of our model is influenced by the pLDDT score from AlphaFold2. The pLDDT score provided by AlphaFold2 serves as an indicator of the accuracy and reliability of the predicted protein structure at a per-residue level. In general, higher pLDDT scores, approaching 100, signify a more accurate and reliable prediction for each residue, and consequently, the predicted results encompass valuable spatial structure information. This wealth of information is expected to contribute to the facilitation of plant vacuole protein identification using our model. Otherwise, it is not conducive to the identification of plant vacuole proteins by our model. Furthermore, our current research may be limited to the identification of organelle proteins. Going forward, we will refine and optimize our methods so that they can be utilized for other protein prediction tasks, including the analysis of primary protein sequences such as protein function, folding, solubility prediction, and drug design.

## Conclusions

This paper proposes a model named GraphIdn, which utilizes the structural characteristics of proteins to identify plant vacuole proteins. The model combines the AlphaFold2 algorithm with a graph convolutional neural network to obtain the structural characteristics of proteins. Through the multi-head attention module, the model learns the weighted contribution of different amino acids in different feature representation subspaces and identifies plant vacuole proteins. The implementation of our model shows superior accuracy in comparison to existing plant vacuole protein (PVP) predictors. The fivefold cross-validation and independent testing have achieved accuracies of 89.93% and 88.51%, respectively. The model has also been successfully extended to identify peroxisomal proteins. The results of the cross-validation show that the GraphIdn model has an accuracy of 94.9% in identifying peroxisomal proteins. This confirms the feasibility of the model and its potential for identifying other organelle proteins.

Of course, there is always room for improvement in our model. In addition to utilizing graph convolutional neural network, we could also explore other graph neural network structures. For protein sequence representation, we could also investigate other methods based on deep representation learning to further enhance the robustness of our model.

Through experiments, we believe that the use of protein structure information is an effective method to improve the performance of sequence-based protein prediction models. More importantly, this architecture could be easily extended to other protein prediction tasks requiring a raw protein sequence.

## Data Availability

The pre-trained ELMo-based SeqVec model and a description on how to implement the embeddings can be found here: https://github.com/Rostlab/SeqVec.The GraphIdn model and datasets can be found here: https://github.com/SJNNNN/GraphIdn.

## Abbreviations

VSR4: Vacuolar sorting receptor 4
VSR6: Vacuolar sorting receptor 6
AVP1: Arabidopsis thaliana vacuolar H +—pyrophosphatase
SAAC: Split amino acid composition
3-gap DPC: 3-Gap dipeptide composition
Bigram PSSM: Bigram position-specific scoring matrix
PseAAC: Pseudo amino acid composition
DC: Dipeptide composition
PsePSSM: Position-specific scoring matrix
EBGW: Evidence-based group work
XGBoost: Extreme gradient boosting
SVM: Support vector machine
DPC: Dipeptide combination (DPC)
K-PSSM: K-spaced position-specific scoring matrix
PSSM: Position-specific scoring matrix
LGBM: Light gradient boosting machine
SFS: Sequential forward search
PDB: Protein data bank
CASP: Critical assessment of protein structure prediction
3D: Three-dimensional
ELMo: Embeddings from language models
SeqVec: Sequence to vector
GaussianNB: Gaussian Naive Bayes
LR: Logistic regression
RF: Random forest
SVM: Support vector machine
LightGBM: Light gradient boosting
GBDT: Gradient boosted decision trees
MLP: Multilayer perceptron
KNN: K-nearest neighbors
Acc: Accuracy
Sn: Sensitivity
Sp: Specificity
MCC: Matthews correlation coefficient
TP: True positive
FP: False positive
TN: True negative
FN: False negative
ROC-AUC: Receiver operating characteristic—area under the curve
PR-AUC: Precision-recall area under the curve
